# Extremely Stable Current Emission of P‐Doped SiC Flexible Field Emitters

**DOI:** 10.1002/advs.201500256

**Published:** 2015-11-17

**Authors:** Shanliang Chen, Minghui Shang, Fengmei Gao, Lin Wang, Pengzhan Ying, Weiyou Yang, Xiaosheng Fang

**Affiliations:** ^1^ Institute of MaterialsNingbo University of TechnologyNingbo City315016P.R. China; ^2^ School of Material Science and EngineeringChina University of Mining and TechnologyXuzhou City221116P.R. China; ^3^ Department of Materials ScienceFudan UniversityShanghai200433P. R. China

**Keywords:** field emitters, flexible devices, low turn‐on fields, P‐doped SiC, stable current emission

## Abstract

**Novel P‐doped SiC flexible field emitters** are developed on carbon fabric substrates, having both low *E*_to_ of 1.03–0.73 Vμm^−1^ up to high temperatures of 673 K, and extremely high current emission stability when subjected to different bending states, bending circle times as well as high temperatures (current emission fluctuations are typically in the range ±2.1%–3.4%).

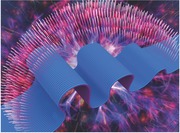

During the past decades, flexible field emission (FE) emitters have attracted considerable interest due to their unique lightweight, conformable, and flexible natures, which offer them the significant advantage to be utilized in flexible FE displays,^1^ e‐papers,^2^ and X‐ray tubes.^3^ Among the families of the field emitters, SiC nanostructures are always considered as one of the hot topics, owing to their excellent physical properties, such as high strength and stiffness, high‐temperature stability, corrosion resistance, and high thermal conductivity.^4^, ^5^, ^6^ Up to date, many efforts have been devoted to the exploration of SiC nanostructured field emitters, suggesting that the SiC 1D counterparts have excellent FE performance with the *E*_to_ (defined to the electric field required to generate a current density of 10 μAcm^−2^), often in several Vμm^−1^,^7^, ^8^, ^9^, ^10^, ^11^ and are very promising to be applied as robust flexible field emitters.^12^, ^13^, ^14^, ^15^ There are mainly three strategies for enhancing the FE performance of SiC 1D nanostructures, i.e., (i) making the nanostructures with sharp tips for utilizing the effect of local electric field,^14^, ^16^ (ii) increasing the density of the emitting sites by surface decoration and making the emitter with rough surface,^17^, ^18^ and (iii) tailoring the band gap of established nanomaterials via doping strategy.^15^, ^19^ However, most of the reported works just focused on lowering the *E*_to_ of the SiC nanostructures, in regard to two main strategies such as enhancing the field enhancement factor (*β*) and reducing the work function (*Φ*) based on the F‐N theory.^10^, ^14^, ^19^, ^20^ There still remains a great obstacle to accomplish a well current emission stability of the desired emitters, which is one of the critically important issues to push forward the practical applications of the SiC nanostructured field emitters.

Currently, as for the exploration of SiC field emitters, high light was shed on 1D nanostructures, due to their intrinsic high aspect ratio, which could greatly enhance the *β* for achieving a significantly reduced *E*_to_.^17^, ^19^, ^20^, ^21^, ^22^, ^23^, ^24^ However, there are inevitable shortcomings for the SiC 1D nanostructures with high aspect ratios (often higher than 50 in the reported works) to be utilized as the emitters, which are caused by current‐induced Joule heat and electrostatic force.^25^, ^26^ The Joule heat at large current‐induced sintering might lead to the emitters' breakdown and/or make the tips merged together, and the strong electrostatic force could split the emitter's tips and/or even tear the emitters due to the limited connected area between the substrate and the 1D nanostructures,^27^, ^28^ which consequently brings a fluctuation in the current emission and even a failure of the devices.^29^, ^30^ In a brief word, although SiC 1D nanostructure with high aspect ratios could be superior to enhance the *β* for obtaining a low *E*_to_, it might be some of contrary to the current emission stability. Thereby, the exploration of novel SiC nanostructures with revolutionary morphologies, which could have well current emission stability also with low *E*_to_, is highly desired.

In the present work, we report the exploration of flexible P‐doped SiC field emitters, aiming to bring them totally excellent FE performances with both high current emission stability and low *E*_to_. The single‐crystalline P‐doped SiC nanoparticles (SiCNPs) with numerous sharp edges and corners are grown on the flexible carbon fabric substrates *via* catalyst‐free pyrolysis of polymeric precursors. The as‐synthesized flexible P‐doped SiCNPs exhibit very low *E*_to_ of 1.03–0.73 Vμm^−1^ in the range of room temperature (RT) ≈673 K. More importantly, they have extremely high mechanical and electrical stability with the typical current emission fluctuations limited in ±2.1%–3.4%, when subjected to different bending states, bending circle times, as well as high temperatures, suggesting their very promising applications in display devices.

**Figure** **1** displays the morphology of the resultant P‐doped SiC nanostructures on the carbon fabric substrate. Figure 1a shows the product under a low magnification, suggesting that the structures of the carbon fabric substrate preserve very well, even after the high‐temperature treatment under 1723 K. The detailed observations of the as‐grown products under higher magnifications (Figure 1b and c) reveal that the surfaces of each carbon fiber of the substrates have been uniform covered by high density nanoparticles. Figure 1d presents the typical closer observation of the grown nanoparticles, disclosing that they have many sharp edges and corners (detailedly shown as the inset in Figure 1d). The sizes of the SiCNPs are 150–300 nm. Figure 1e shows the interface between the SiCNPs and the carbon fiber substrate. In our experiments, the SiCNPs can be hardly detached from the carbon fabric, implying that the nanoparticles attach the carbon fabric substrate solidly. It could be attributed to the fact that the used carbon fabrics act not only as the substrate but also as some part of the reactants during the pyrolysis process (providing some part of the C sources),^11^ leading to the well‐attached growth of SiCNPs around the carbon fibers (as schematically illustrated in Figure 1f). As compared to the conventional nanowire‐shaped field emitters, the as‐grown SiCNPs with numerous sharp edges and corners could provide much more efficient FE sites due to the local field enhancement effect (as schematically illustrated in Figure 1g). For comparison, the fabrication of pure SiC counterparts is also performed without introducing FePO_4_. It seems that the similar structures of the SiCNPs have grown (Figure S2, Supporting Information), suggesting that, in current case, the P dopants play no influence on the growth of the SiCNPs in morphologies.

**Figure 1:**
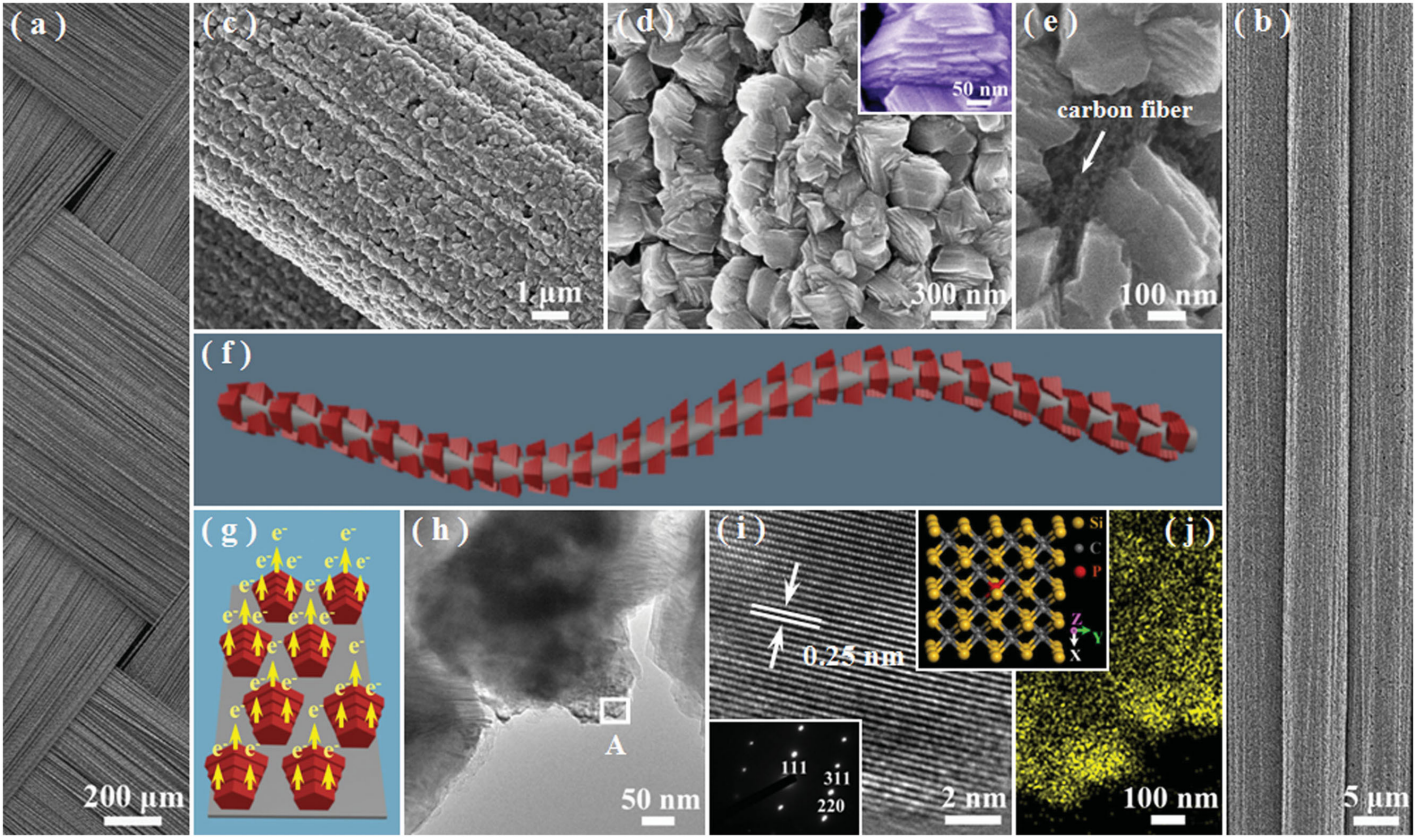
a–e) Typical SEM images of as‐synthesized P‐doped SiCNPs on the carbon fabric substrates under different magnifications. f) Schematic illustration of SiCNPs growth on the carbon fiber surface. g) Schematic illustration for the electron emission from SiCNPs with sharp edges and corners. h) Typical TEM image of SiCNPs. i) The corresponding HRTEM image of the P‐doped SiCNPs recorded from the marked areas of A in panel (h) (the inset is the corresponding SAED pattern). j) Representative element mapping of the P dopants within the SiCNPs. The inset between (i) and (j) shows the crystal structure of SiC with the substitutional solid solution of P dopants.

The microstructures of the P‐doped SiCNPs are further characterized by transmission electron microscopy (TEM). Figure 1h shows a typical TEM image of the SiCNPs under a high magnification, confirming the formed rough surface of the resultant nanoparticles with sharp edges and corner, whose radius of curvature are often sized in tens of nanometers. These numerous sharp edges and corners could be acted as the efficient electron emission sites, which could greatly enhance the electron emission ability of the nanoparticles (as schematically illustrated in Figure 1h), owing to the local field effects.^20^ Both the high‐resolution TEM (HRTEM) image (Figure 1i) and the corresponding selected area diffraction (SAED) pattern (the inset in Figure 1i) recorded from the marked area of A in Figure 1g indicate the single‐crystalline nature of a single SiC nanoparticle. The interplanar spacing *d* of two neighbored lattice fringes is ≈0.25 nm, fitting the {111} plane distance of 3*C*‐SiC, suggesting the as‐synthesized SiCNPs grow along the <111> direction, which is the preferred one of 3*C*‐SiC to maintain the minimum surface energy of the crystal.^11^, ^31^, ^32^ The growth of SiCNPs should be dominant by a typical vapor–solid process, since no catalysts are introduced, which is discussed in Figure S3 in the Supporting Information. The growth of numerous sharp edges and corners around the surface of the SiC nanoparticles could be attributed to the incomplete process of surface energy minimization of the laterally faceted crystal planes via atomic diffusion.^18^, ^33^ Figure 1j qualitatively shows a typical element mapping of P, suggesting that the P dopants are richer on the edge/surface region of the SiCNPs. The detailed X‐ray diffraction (XRD), X‐ray photoelectron spectroscopy (XPS), and Raman characterizations^34^, ^35^ verify that the P dopants have been successfully incorporated into the lattice of the SiCNPS with a typical concentration of >>0.27 at% (see Figure S4, Supporting Information).

The FE characteristics of as‐synthesized pure and P‐doped SiCNPs under room temperature (RT, ≈298 K) are revealed by FE current density (*J*_f_) versus the applied electric field (*E*) plots, as shown in **Figure** **2**a (the experiment setup, see Figure S5, Supporting Information). The *J*_f_–*E* plots are both relatively smooth and consistent, indicating their stable electron emission. The FE *J*_f_–*E* characteristic of the pure and P‐doped SiCNPs are analyzed on the basis of classical Fowler–Nordheim (F‐N) theory^36^

(1) $$J_{f}=(A\beta^{2}E^{2}/\Phi )\cdot \exp(-B\Phi^{3/2}/\beta E)$$

Where *E* is the applied electric field, *A* = 1.54 × 10^−6^ AeVV^−2^, *B* = 6.83 × 10^3^ eV^−3/2^ Vμm^−1^, *β* is the field enhanced factor, and *Φ* is the work function of the emitter material (i.e., 4.0 eV for SiC^6^. The corresponding F‐N plots, obtained by plotting ln(*J*/*E* ^2^) versus1/*E*, are presented in Figure S6a, Supporting Information. The linear relationships of the F‐N plots indicate that the electron emissions from the pure and P‐doped SiCNPs both follow the conventional FE mechanism. According to the slopes of the F‐N plots, the field enhancement factors (*β*) of the pure and P‐doped SiCNPs are ≈1283 and 5508, respectively. Note that the *β* of P‐doped SiCNPs is larger than most of those ever reported in the previous works on the SiC nanostructure‐based emitters, other typical nanostructure‐based flexible emitters as well as the other commonly used emitters. (see Table S1, Supporting Information). According to the *J*_f_ *–E* plots of these two samples, the *E*_to_ and threshold fields (*E*_thr_, defined to the electric field required to generate a current density of 1 mAcm^−2^) of P‐doped and pure SiCNPs are 1.03, 2.96 Vμm^−1^ and 1.49, 3.70 Vμm^−1^, respectively, as shown in Figure 2b and Figure S6b, Supporting Information, respectively. These suggest, compared with the pure SiCNPs, that the *E*_to_ and *E*_thr_ of the P‐doped ones have been reduced by nearly three times, and more than two times, respectively. With respect to their similar morphologies between the pure and P‐doped SiCNPs (Figure 1 and Figure S2, Supporting Information), the enhanced FE behaviors should be mainly attributed to the P dopants, suggesting that the P dopants could bring a significant improvement on the FE properties of SiCNPs. It also should be pointed out, compared to the previously reported works on the FE of SiC nanostructure‐based emitters as well as other typical nanostructure‐based flexible emitters, the *E*_to_ and *E*_thr_ of our P‐doped SiCNPs could be comparable to the lowest one ever reported (see Table S1, Supporting Information), suggesting their very excellent FE properties. This could be mainly attributed to the following two reasons: First, the numerous sharp edges and corners around the surfaces of the P‐doped SiCNPs could be serviced as the efficient emitter sites, which can greatly enhance the electron emission because of the highly increased density of emission sites as well as the strong local field effects.^16^, ^18^, ^19^, ^20^ Second, in line with the reported works concerning on the enhanced FE of SiC 1D nanostructures with N/B/Al dopants,^12^, ^13^, ^15^, ^19^, ^37^, ^38^ the P dopants within the SiCNPs could play a profound contribution to the enhanced FE behaviors, by favoring the formation of localized impurity states near the conduction band edge and reducing the work function (Figure S7, Supporting Information). As compared to the previous works of Al/B/N‐doped SiC nanowire field emitters (the Al/B/N doping often falls in several at% level),^12^, ^13^, ^14^, ^15^, ^16^, ^19^, ^37^, ^38^, ^39^ the P‐doping level in current case is an order of magnitude lower (0.27 at%), suggesting that the P dopants are much more effective to create the localized impurity states near the conduction band edge and reduce the work function. Another important case should be noted that the aspect ratio of current SiCNPs (nearly be spherical) is much lower than those of the nanowires (often higher than 50).^12^, ^13^, ^15^, ^19^, ^37^ The larger aspect ratio of the nanostructures could offer a better FE property of the emitters, since it can enhance the field enhancement factor.^40^, ^41^ Considering the *E*_to_ could be comparable to the lowest one of SiC low‐dimensional nanostructured with high aspect ratios ever reported (see Table S1, Supporting Information), it further verifies that the P dopants could be much more effective to enhance the electron emission of SiC emitters.

**Figure 2:**
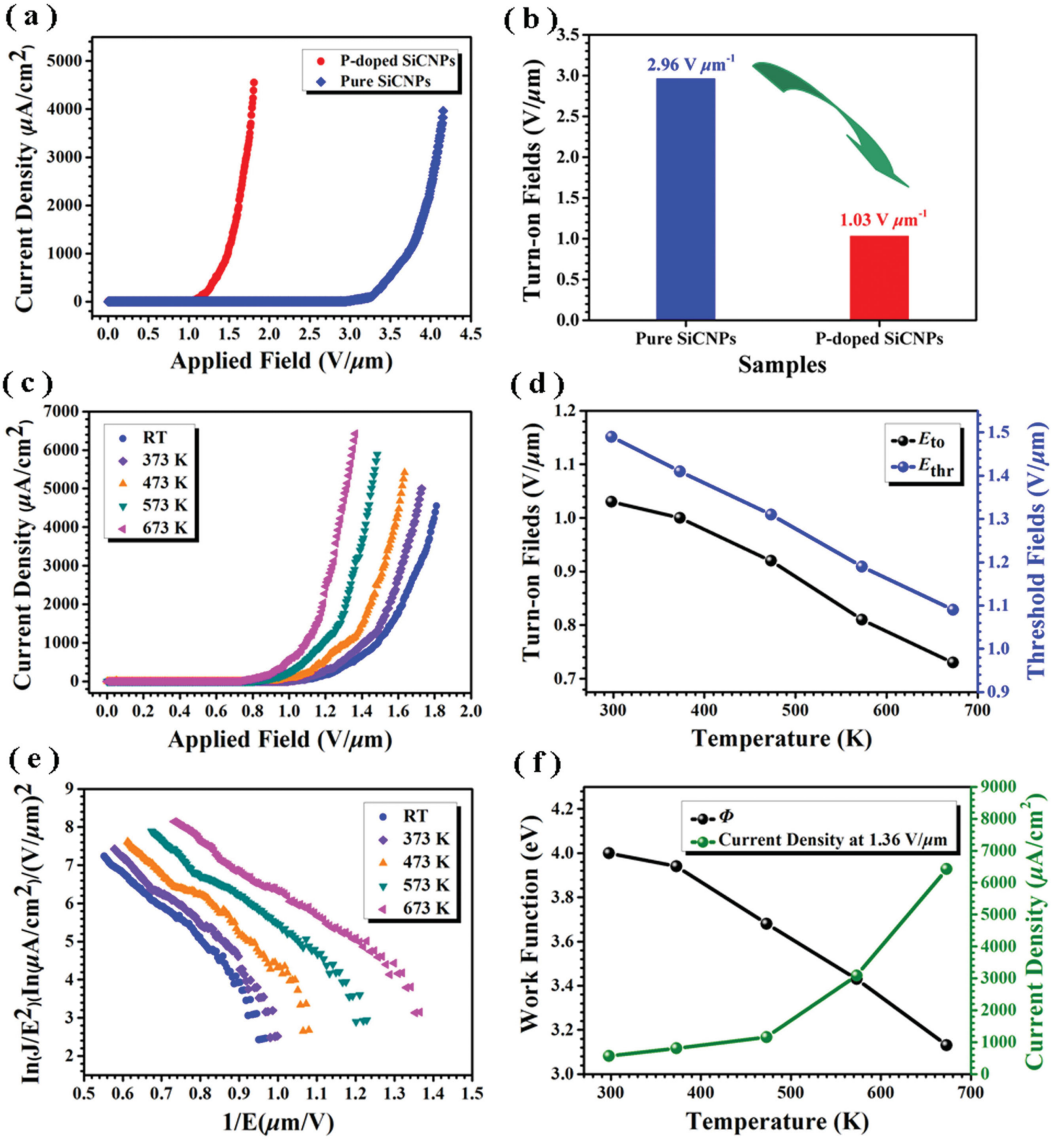
a) *J*_f_–*E* curves of pure and P‐doped SiCNPs under RT. b) The variations of *E*_to_ between pure SiCNPs and P‐doped counterparts. c) *J*_f_ *–E* curves of P‐doped SiCNPs under different temperatures. d) The variations of *E*_to_ and *E*_thr_ with the change of the temperatures. e) The corresponding F‐N plots. f) The variations of work functions (*Φ*) and emission current densities at 1.36 Vμm^−1^ with the change of the temperatures.

Figure 2c depicts the *J–E* plots of the selected P‐doped SiCNPs at different temperatures in the range of RT ≈673 K. The smooth and consistent curves confirm the stable electron emission under the given high temperatures. Accordingly, the *E*_to_ and *E*_thr_ are 1.03, 1.0, 0.92, 0.81, and 0.73 V μm^−1^ and 1.49, 1.41, 1.31, 1.19, and 1.09 Vμm^−1^ with the increase in temperatures from RT to 673 K, respectively, as shown in Figure 2d, respectively. For the temperature‐dependent FE behaviors, the total current density (*J*) should be often contributed from the field current density (*J*_f_) and thermionic current density (*J*_t_).^42^, ^43^, ^44^ However, for the field emitter cathode materials with work functions of 3–5 eV and serviced below 1000 K, the contribution from thermionic emission is much smaller than that of the FE.^43^, ^45^, ^46^ This could be also confirmed by the present experimental results. As shown in Figure 2c, the emission current densities are all nearly 0 over the applied fields ranged from 0 to ≈0.7 Vμm^−1^ under RT ≈673 K, suggesting that the FE contribution from thermionic electron emission is very tiny to be ignored. To verify the point, the F‐N equation is used to examine the electron tunneling phenomena, as shown in Figure 2e. The approximately linear relationships of the F‐N plots further confirm that the P‐doped SiCNPs all obey the conventional field electron emission mechanism, regardless of the fixed different temperatures. With regard to no significant influence of the temperatures bellow 673 K on the intrinsic crystal structure of SiC,^47^ the *β* can be considered as a constant, regardless of the temperature variations. By tracing the F‐N plots and taking the work function of 4.0 eV for SiC at RT,^6^ the *Φ* could be calculated by Equation (2)

(2) $$k_{\mathrm{slope}}=-B\Phi^{3/2}/\beta$$

which is shown in Figure 2f. It is revealed that the *Φ* is decreased with the increase in temperatures. In addition, benefited from the decreased *Φ*, the emission current densities show a significant increase upon the increase in temperatures. For example, the current density suggests a significant increase from 562 to 6425 μA cm^−2^ at 1.36 V μm^−1^ with the increase in temperatures from RT to 673 K, which is two‐order‐of‐magnitudes higher than that at RT (Figure 2f). The temperature‐dependent FE behaviors could be mainly ascribed to the increase of the electron–hole pairs caused by the increase in temperatures.^37^, ^48^, ^49^

As for ideal flexible field emitters, besides the required low *E*_to_ and *E*_thr_, the mechanical and electrical stability are also highly desired for practical device applications. Thus, we now come to the point about the mechanical stability of the as‐synthesized P‐doped SiCNPs flexible field emitters against the repeated bending cycles, bending states, and high temperatures. **Figure** **3**a depicts the digital photographs of P‐doped SiCNPs flexible field emitters, which can be strongly twisted periodically without noticeable structural destruction, suggesting the high flexibility and robust of the as‐synthesized field emitters. Figure 3b shows the current emission stabilities under different repeated bending cycles up to 200 times with a bending radio of ≈1.2 cm. The obtained *J*_f_ *–E* curves are relatively smooth and consistent, and the variations of *E*_to_ are nearly negligible, suggesting the highly mechanical stability of the obtained SiCNPs emitters. The corresponding linear F‐N plots are also similar and follow the conventional F‐N rule, as shown in Figure 3c. Interestingly, compared with the SiC nanostructures in Figure 1(a–e) before bending, there are nearly no damage in structures happened after 200 repeated bending cycles (Figure S8, Supporting Information), further demonstrating the mechanical robust of the present P‐doped SiCNPs emitters. Figure 3d shows the *J*_f_–*E* plots of the P‐doped SiCNPs field emitters under the states of flat, convex and concave configuration (the bending is fixed at ≈1.2 cm in radio for both convex and concave geometries). It is found that, different from the reported 1D nanostructured flexible field emitters always exhibiting distinctively different FE behaviors under various bending states,^15^, ^50^, ^51^, ^52^ the *E*_to_ and *E*_thr_ are almost identical to the flat one, regardless of the various bending states. The linear F‐N plots are also quite similar (Figure 3e), implying that the present P‐doped SiCNPs are very effective to limit the “screening effect.”^51^, ^53^

**Figure 3:**
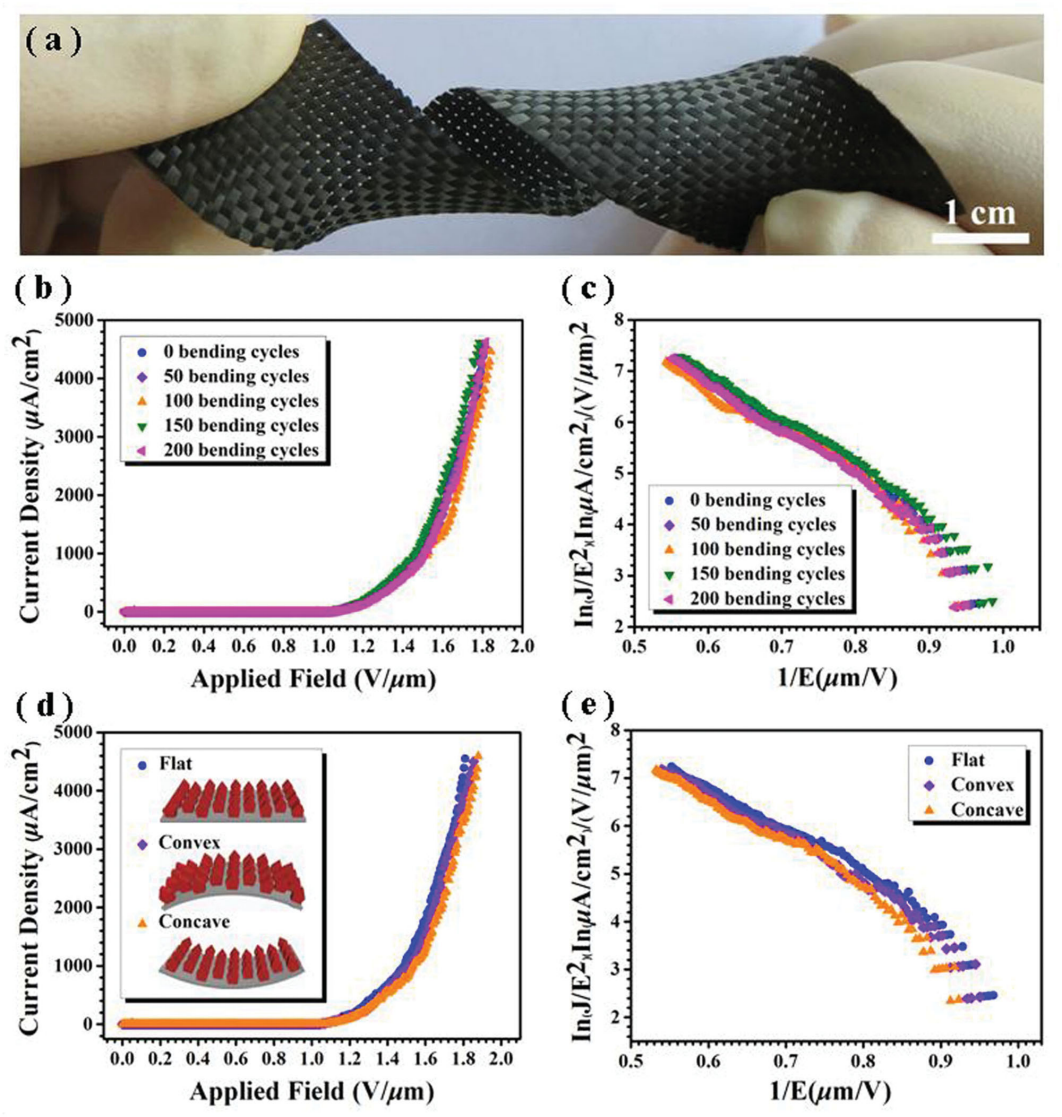
a) A typical digital photo showing the highly flexibility of the P‐doped SiCNPs field emitters. b) *J*_f_ *–E* curves of P‐doped SiCNPs after 0, 50, 100, 150, 200 bending cycles. c) The corresponding F‐N plots. d) *J*_f_ *–E* curves of P‐doped SiCNPs under different bending states. e) The corresponding F‐N plots under different bending states.

The current emission stabilities of the as‐synthesized P‐doped SiCNPs flexible emitters are also measured in the range of RT to 673 K. As shown in **Figure** **4**a, during 20 h of the continuous operation at a current density of ≈2650 μAcm^−2^ under RT, the current density fluctuation is only ±3.0% with no trace of current density degradation, which is the lowest one among those of the reported works on 1D SiC emitters, other typical nanostructure‐based flexible emitters as well as other commonly used emitters (see Table S1, Supporting Information). Meanwhile, no any structure damaged of the P‐doped SiCNPs field emitters after the long working time of 20 h, as shown in Figure 4b. Figure 4c presents the current emission stabilities of the P‐doped SiCNPs emitters at a flat state over 4 h in ≈2650 *μ*Acm^−2^ under different temperatures (RT ≈ 673 K). There are no degradation in current density under the given work temperatures at RT, 373, 473, 573, and 673 K, and their corresponding current emission fluctuations are ≈±3.0%, ±2.7%, ±2.9%, ±3.0%, and ±3.4%, respectively, suggesting that the P‐doped SiCNP emitters could be well serviced up to 673 K. Figure 4d shows the current emission stabilities of the as‐synthesized P‐doped SiCNPs under different bending states over 4 h in ≈2650 μAcm^−2^. No degradation in current density can be observed for each state, and the current emission fluctuations are ≈±3.0%, ± 2.6%, ±2.5%, ±2.1%, and ±2.8%, as for the SiCNPs field emitters configured in flat state, convex bending with the radios of ≈1.2 and ≈0.4 cm, concave bending with the radios of ≈1.2 and ≈0.4 cm, respectively, indicating the stresses of the substrate caused by bending do not have significant effect on the current emission stabilities. These experimental results strongly represent that our P‐doped SiCNPs flexible field emitters are highly robust with excellent mechanical and electrical stabilities.

**Figure 4:**
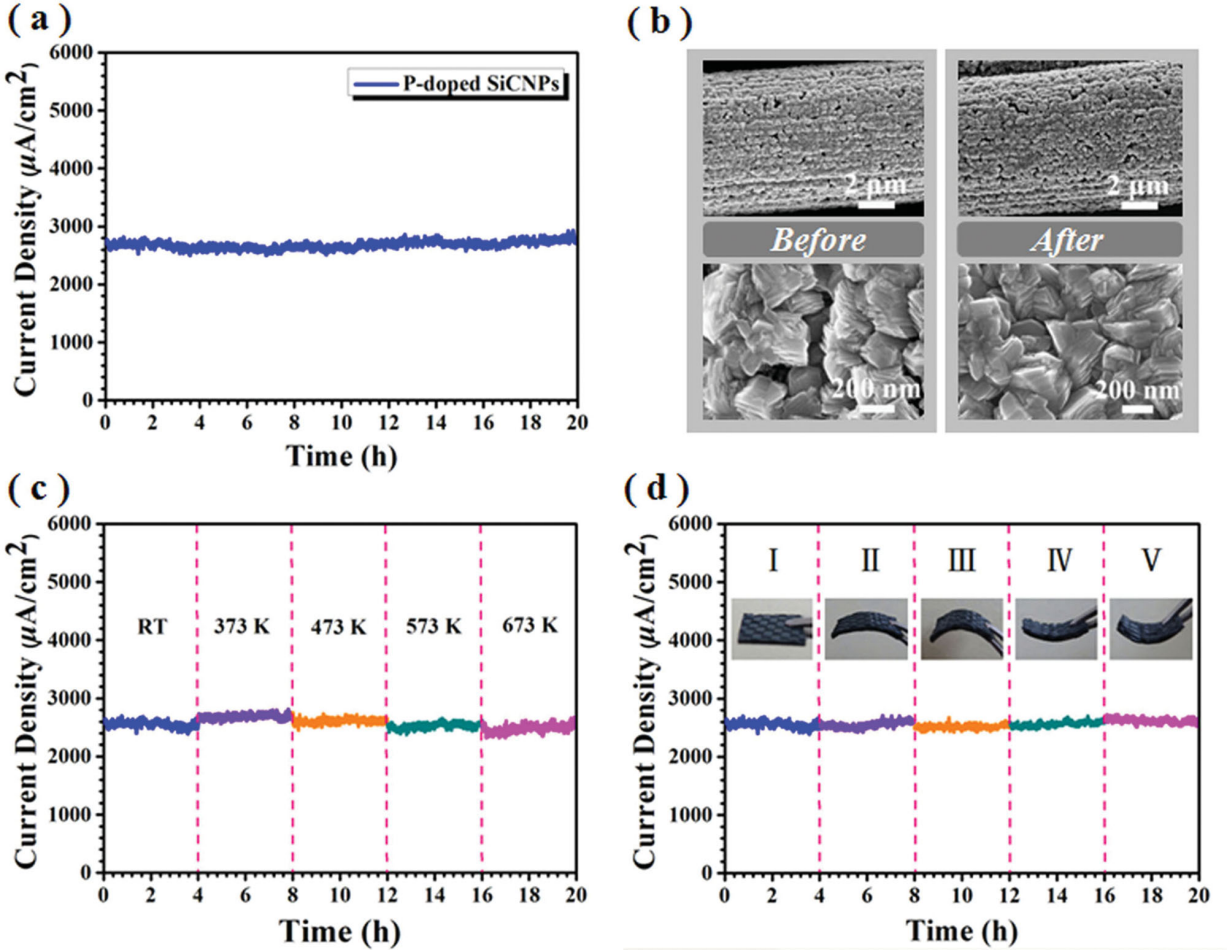
a) The current emission stability of P‐doped SiCNPs over 20 h at the emission current density of ≈2650 μAcm^−2^ under RT. b) The typical SEM images of P‐doped SiCNPs before and after 20 h FE operation. c) The current emission stabilities of flexible P‐doped SiCNPs under different temperatures. d) The current emission stabilities of the flexible P‐doped SiCNPs under each bent state over 4 h (I: in flat shape; II and III: in convex shape with radii of ≈1.2 and 0.4 cm, respectively; IV and V: in concave shape with radii of ≈1.2 and 0.4 cm, respectively).

The mechanical and electrical stability of the field emitters should be treated carefully for their device applications, especially once they fall into nanodimensional/microdimensional scale. As compared to their bulk counterparts, the nano‐/micro‐sized emitters possess larger surface areas, lower melting points, and larger scale bending, which make them be vulnerable to against chemical and physical damages.^27^, ^28^, ^54^ According to previously reported works on individual 1D nanostructured emitters, the failure of field emitters can be mainly considered as a combination of two effects, namely the Joule heating and electrostatic force.^26^, ^28^, ^29^ The Joule heat generated during FE process might cause the emitter initially degraded at the maximum temperature followed by segment breakdown from the 1D emitter's tip.^27^ Meanwhile, the electrostatic force on the emitter surface would generate the Maxwell stress, which can be further divided into axial stress and radial stress.^27^ The radial stress might cause the emitter's tip split,^30^, ^55^ while the axial stress would break and tear away the emitter from the substrate.^25^, ^29^ Herein, in current case, the totally excellent mechanical and electron emission stability of the P‐doped SiCNPs could be mainly attributed to following points: (i) The high stable morphology of the SiCNPs. The SiC nanoparticles with numerous sharp edges and corners possess low aspect ratios (Figure 1d–e), which ensure the emitters to be robust against the emitter's tip breakdown caused by the Joule heat effect and splitting induced by the radial stress of electrostatic force effect.^28^, ^56^ (ii) The well connection between the carbon fabric substrate and SiCNPs. In our case, the connected area between the SiCNPs emitter and the substrate is much larger than those between the 1D nanostructures and the substrate. As reported by Tang et al., a large connection area between the emitters and the substrate can facilitate the transfer of the Joule heat from the tip to the Si substrate. Accordingly, the tip can be effectively protected from being destroyed due to the superheat, leading to a good thermal stability of the field emitters.^57^ Also, the strong combination benefited from the large connected area between the bases of SiC nanotips and the carbon fiber substrate can guarantee the tips not be destroyed from the substrate caused by the axial stress of the electrostatic force effect, resulting in the little change of the emission sites^58^, ^59^ (see Figure 1e and Figure S2f, Supporting Information). (iii) The low aspect ratio of the field emitters. The as‐synthesized SiCNPs field emitters possess much lower aspect ratio as compared to those of the 1D nanostructures often with a aspect ratio higher than 50. The current emission fluctuations are ≈±3.0%, ± 2.6%, ±2.5%, ±2.1%, and ±2.8%, as for the SiCNPs field emitters configured in flat state, convex bending with the radios of ≈1.2 and ≈0.4 cm, concave bending with the radios of ≈1.2 and ≈0.4 cm, which suggest the significant enhanced current emission stabilities as compared to those of ≈8.9%, 8.1%, and 7.1% responding to the concave, flat and convex states of the N‐doping SiC nanoneedles, respectively.^15^ It indicates that the stresses of the substrate caused by bending do not have significant effect on the current emission stabilities. As for flexible device application, a lower aspect ratio of the emitters could favor a more stable electron emission direction against the change of the bending states, which consequently limit the “screening effect,” as schematically illustrated in Figure S9, Supporting Information. (iv) The P dopants. In terms of the reported current emission fluctuations, the 1D SiC nanostructures (e.g., at RT and 473 K, those of pure SiC nanowires are 9% and 22%;^18^ those of the Al‐doped SiC nanowires are 14% and 11%;^18^ those of the B‐doped SiC nanoarrays are ≈6.5% and 7.8%;^38^ and those of the N‐dopant SiC nanoneedles are 7.7% and 14.1%, respectively^37^), the current emission fluctuations of the P‐doped SiCNPs emitters are 3.0%, 2.7%, 2.9%, 3.0%, and 3.4% at the given high temperatures at RT, 373, 473, 573, and 673 K. The significant reduced current emission fluctuations and remarkably decreased differences between working at RT and high temperatures suggest that, as compared to the Al/B/N doping of SiC nanostructures, the incorporated P dopants could be much more effective to enhance the FE behaviors with excellent current emission stabilities.

The FE performances of our P‐doped SiCNP emitters (including the *E*_to_, *E*_thr_, field enhancement factors (*β*), maximum FE current density, and FE current stability), compared with those of the representative 1D SiC nanostructured field emitters, other typical nanostructured flexible emitters as well as other commonly used emitters, are summarized in Table S1 (Supporting Information) to show the state‐of‐the‐art progress of the field emitter. It suggests the points as below: (i) The *E*_to_ (0.73–1.03 Vμm^−1^) and *E*_thr_ (1.09–1.49 Vμm^−1^) of the present P‐doped SiCNPs emitters could be comparable to the lowest one of the nanostructured field emitters ever reported. (ii) The field enhancement factor (*β*) of P‐doped SiCNPs is calculated to be >>5508, which is larger than most of the reported emitters. (iii) The maximum emission current density of 6600 μA/cm^2^ was achieved at 1.36 Vμm^−1^ under 673 K. Although it is lower than those in some of the reported work (e.g., that of the ultrathin ZnO nanobelt emitters is up to 40.17 mAcm^−2^)^60^, this value is higher than most of the reported 1D SiC nanostructured field emitters, other typical nanostructured flexible emitters, as well as other commonly used emitters. (iv) The current emission fluctuation of P‐doped SiCNPs is measured to be just ±2.1%–3.4%, which is lower than most of the typical emitters. It is worthy noting that the current fluctuations of our SiCNPs emitters are recorded under the harsh working conditions, such as the high temperatures, various bending states, and different bending cycles, suggesting their robust mechanical and electrical stability, which have not been achieved ever before. These experimental results demonstrate that the present P‐doped SiCNP field emitters exhibit the significant advantage among the commonly used nanostructured emitters, implying their very promising potential for practical application in flexible FE devices, especially for those serviced under harsh work conditions.

In summary, we have reported the exploration of flexible SiC field emitters, which could have both low *E*_to_ and extremely high current emission stability. The P‐doped SiCNPs were uniformly grown on the carbon fabric substrates via a catalyst‐free pyrolysis of polymeric precursors. The as‐synthesized P‐doped SiCNP emitters exhibit low *E*_to_ of 1.03–0.73 Vμm^−1^ with the temperatures raised from RT to 673 K. The current emission fluctuations of the P‐doped SiCNP emitters are typically of ±2.1%–3.4%, when subjected to different bending states, bending circle times, as well as high temperatures. The totally excellent FE performances could be attributed to the desired structure characteristics of the as‐synthesized SiCNPs, such as with numerous sharp edges and corners, incorporated P dopants, very low aspect ratios as well as large connected interface areas. It is believed that the present work could bring a breakthrough to the exploration of novel flexible SiC field emitters, which would push forward them to be practically applied in flexible FE nanodevices.

## Experimental Section

The pure and P‐doped SiC nanoparticles were synthesized by catalyst‐free pyrolysis of polysilazane (PSN (Figure S1, Supporting Information), Institute of Chemistry, Chinese Academic of Science, China) in a graphite‐heater furnace, which were commercially available and used directly without further purification. The PSN was first solidified by heat‐treatment at 533 K for 30 min under Ar atmosphere, and then subjected to be ball‐milled into fine powders. The carbon fabric substrate was placed on the top of a graphic crucible (purity: 99%). The raw materials of 0.3 g PSN or 0.3 g PSN + 0.06 g FePO_4_·4H_2_O (Adamas, Shanghai, China) mixed powders were used for the growth of pure and P‐doped SiC nanostructures, respectively. Then the crucible containing the powders with the substrate was placed into the graphite‐heater furnace. The furnace chamber was first pumped to 10^−4^ Pa and followed by introducing Ar (99.99%, 0.1 Mpa) into the chamber to reduce O_2_ to a negligible level. Subsequently, the system was heated up to the desired temperature of 1723 K at a rate of 30 K min^−1^, and then cooling down to 1373 K at a cooling rate of 23 K min^−1^, followed by furnace cool to RT. The whole pyrolysis process was carried out under the argon (99.99%, 0.1 Mpa) at a flowing rate of 200 sccm.

The obtained products were characterized using field emission scanning electron microscopy (S‐4800, Hitachi, Japan), X‐ray diffraction (XRD, D8 Advance, Bruker, Germany) with Cu Kα radiation (*λ* = 1.5406), high‐resolution transmission electron microscopy (HRTEM, JEM‐2100F, JEOL, Japan) equipped with energy dispersive X‐ray spectroscopy (EDX, Quantax‐STEM, Bruker, Germany) and X‐ray photoelectron spectroscopy (XPS, AXIS ULTRA DLD, Shimadzu, Japan). The FE properties of the SiCNPs were performed on a home‐built high vacuum FE setup with a base pressure of ≈1.5 × 10^−7^ Pa at RT. The current–voltage curves were recorded on a Keithley 248 unit with a detection resolution of 0.1 fA. The distance between the surface of SiC nanostructures and the anode of vacuum chamber was typically fixed at ≈800 μm.

## Supporting information

As a service to our authors and readers, this journal provides supporting information supplied by the authors. Such materials are peer reviewed and may be re‐organized for online delivery, but are not copy‐edited or typeset. Technical support issues arising from supporting information (other than missing files) should be addressed to the authors.

Supplementary
