# Supplementary material for: Extremely Stable Current Emission of P‐Doped SiC Flexible Field Emitters
Source: Adv Sci (Weinh). 2015 Nov 17;3(1):1500256. doi: 10.1002/advs.201500256 (PMC5063129; doi:10.1002/advs.201500256)
Supplement: Supplementary file 1 — Supplementary [file ADVS-3-0j-s001.pdf]

Copyright WILEY-VCH Verlag GmbH & Co. KGaA, 69469 Weinheim, Germany, 2015.

Supporting Information

for *Adv. Sci.*, DOI: 10.1002/advs.201500256

Extremely Stable Current Emission of P-Doped SiC Flexible Field Emitters

Shanliang Chen, Minghui Shang, Fengmei Gao, Lin Wang, Pengzhan Ying, Weiyou Yang, and Xiaosheng Fang**

Supporting Information

Extremely Stable Current Emission of P-doped SiC Flexible Field Emitters

By *Shanliang Chen, Minghui Shang, Fengmei Gao, Lin Wang, Pengzhan Ying, Weiyou Yang* and Xiaosheng Fang**

[*] Dr. S. L. Chen, Dr. M. H. Shang, Dr. F. M. Gao, L. Wang, Prof. W. Y. Yang

Institute of Materials

Ningbo University of Technology

Ningbo City, 315016, P.R. China

E-mail: weiyouyang@tsinghua.org.cn (W. Y. Yang)

Dr. S. L. Chen, Dr. P. Z. Ying

School of Material Science and Engineering

China University of Mining and Technology

Xuzhou City, 221116, P.R. China.

Prof. X. S. Fang

Department of Materials Science

Fudan University

Shanghai 200433 (P. R. China)

E-mail: xshfang@fudan.edu.cn (X. S. Fang)

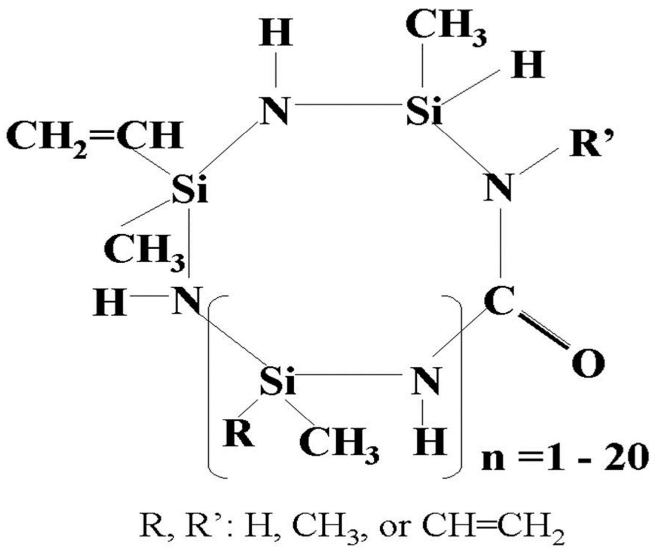

Figure S1. Molecular structure of the precursors polysilazane (PSN).

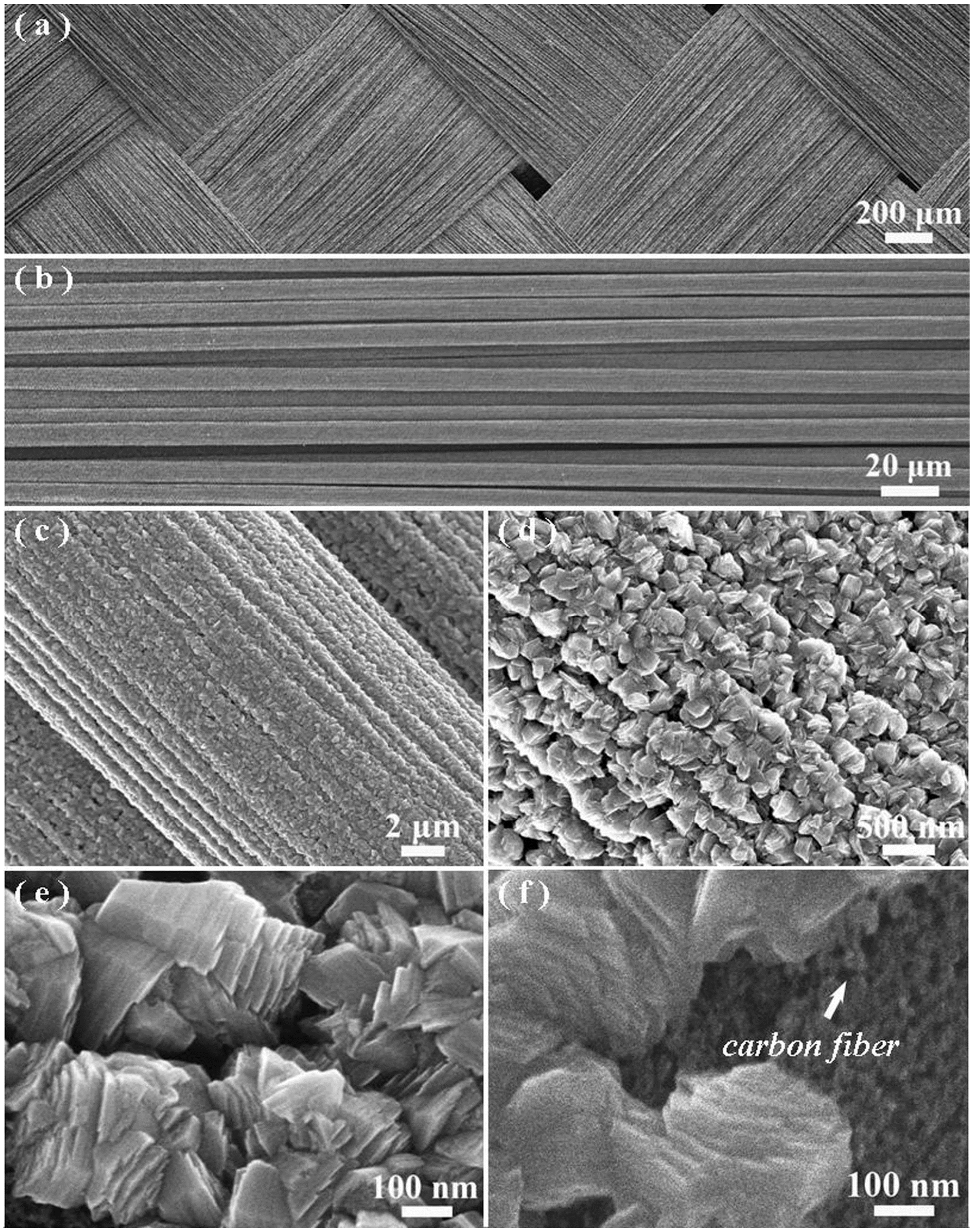

Figure S2. (a-f) Typical SEM images of as-synthesized pure SiC nanoparticles under different magnifications.

Proposed Mechanism for the Growth of P-doped SiCNPs

With respect to no metal catalysts used in our case, the growth of the P-doped SiCNPs should be dominated by the typical Vapor-Solid (VS) process, which is schematically illustrated in **Figure S3**. During the heating process, the PSN polymeric precursors (their molecular structure are shown as Figure S1) will be converted into metastable SiCN amorphous ceramics with a small amount of O when the temperature exceeds 1000°C,[1] followed by releasing the vapor phases of SiO and CO with the further increase of the temperatures.[2] The reaction of SiO and C (*i.e.*, carbon fabrics of the substrate) by Reaction (1) (step I in Figure S3) will make the nucleation of SiC on the substrates of the carbon fabrics, accompanying by the release of O_2, which favors the oxidation of the SiCN to the vapors of SiO and CO. The reaction of SiO and CO (Reaction (2)) will make the deposition of SiC on the preformed SiC nucleus, thus leading the continuous growth of SiC nanocrystals (Step II-V). At the meaning time, the CO_2 will react with the C, which is mainly derived from the raw materials of SiCN and the carbon fabric substrate, to form CO under the used high temperature of 1723 K (Reaction 2) to facilitate the Reaction (2) to be proceeded, resulting the growth of the SiCNPs (Step II-V). The growth of numerous sharp edges and corners around the surface of the SiC nanoparticles could be attributed to the incomplete process of surface energy minimization of the laterally faceted crystal planes *via* atomic diffusion.[3,4]

$$2SiO_{(g)} + 2C_{(s)} = 2SiC_{(s)} + O_{2(g)} \quad \ldots\ldots\ldots\ldots\ldots\ldots (1)$$

$$SiO_{(g)} + 3CO_{(g)} = SiC_{(s)} + CO_{2(g)} \quad \ldots\ldots\ldots\ldots\ldots\ldots (2)$$

$$CO_{2(g)} + C_{(s)} = 2CO_{(g)} \quad \ldots\ldots\ldots\ldots\ldots\ldots \text{...........}(3)$$

There are mainly two possible atoms doping mechanisms for the SiC nanostructures:[5,6] one is through the formation of a substitution solid solution, and the other is via the formation of an interstitial solid solution. Based on the detailed examination of the typical XRD pattern of the as-synthesized SiCNPs, the (111) peak of P-doped SiCNPs shifts to a higher angle with a $\Delta 2\theta$ of 0.102°, suggesting that the doped P atoms into the SiC crystal lattice should be dominated by the formation of substitution

solid solutions. It is known that the atomic radius of Si, C and P are 0.117, 0.110 and 0.077 nm, respectively. It seems that the atomic radius of P is comparable to that of Si, and much bigger than that of C. Thus, the substitution of P to Si should be much easier than the substitution of P to C, due to the much fewer energy required to be overcomed induced by the doping derived lattice distortion.[7]) In a brief word, the doping of P into the SiC crystals should be performed by the formation of solid solutions via the substitution of P to Si.

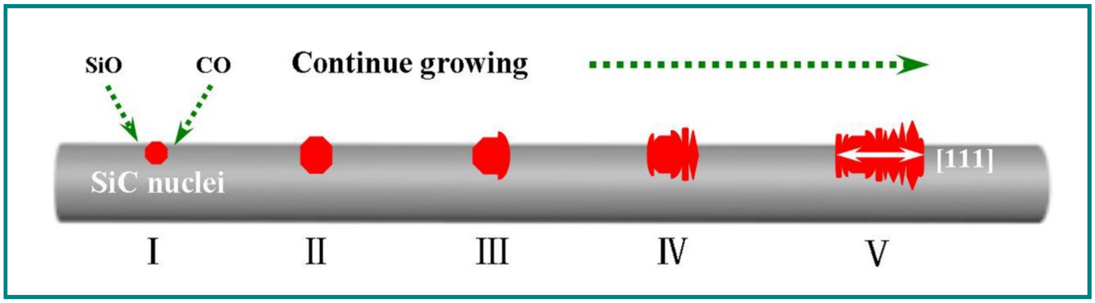

Figure S3. The proposed growth mechanism of SiCNPs on the carbon fabric substrate.

XRD, XPS and Raman Spectra Characterizations of P-doped SiCNPs

Figure S4a provides the typical XRD patterns recorded from the pure and P-doped SiCNPs on carbon fabric substrate. Beside the detected signals from the carbon fabric substrate, all the other peaks match the 3*C*-SiC (JCPDS Card No. 29-1129) well, implying that the as-grown nanostructures are pure 3*C*-SiC phase. The strong and sharp diffraction peaks indicate that the SiCNPs are high crystallinity. The low intensity peak marked with “S.F.” is attributed to the stacking faults within the 3*C*-SiC crystals.[8] Figure S4b presents a closer examination of the (111) peak of the XRD patterns. Compared to that of pure SiCNPs, the center of (111) peak of P-doped SiCNPs shifts to a higher angle with a $\Delta 2\theta$ of 0.102°. The calculated lattice parameter (a) shows a 0.43% decrease as compared with that of the pure SiC, confirming that the P atoms have been successfully doped into the nanoparticles *via* the formation of substitutional solid solutions (*i.e.*, the substitution of P to Si atoms, as schematically shown in Figure S4c).[5-7]

To further provide the evidence for the P doping of the SiC nanoparticles, X-ray photoelectron spectroscopy (XPS) is employed to disclose the compositions of the P-doped SiCNPs. The binding energies obtained in the XPS spectra are standardized for specimen charging using C 1s as the reference at 284.6 eV. Figure S4c and S4d respond to the XPS spectra of Si and C, respectively. The peaks centered at 102.8 and 284.8 eV are given rise to the binding energies of Si 2p and C 1s of SiC, respectively. The Si 2p peak with asymmetric shape and high binding energy tailing suggest the existence of SiO_x species around the SiC surface.[9] The C 1s spectra also displays another peak at a higher binding energy (~286.1 eV), which can be ascribed to the adsorbed CO at the nanoparticles surface during the pyrolysis of polymeric process. Notably, the P 2p peak at ~125.8 eV is detected (Figure S4e), clearly verifying the P dopants within the SiCNPs. Accordingly, the concentration of the P dopants is ~0.27 *at.*%.

The typical Raman spectra of the pure and P-doped SiCNPs are recorded with a wave laser of 633 nm as the excitation source, which are shown in Figure S4f. Both of the two types SiCNPs exhibit two

broad absorption bands, respectively. It seems that the maximum values of pure SiCNPs appear at ~790 and 923 cm^{-1}, whereas those of P-doped SiCNPs shift to ~786 and 921 cm^{-1}. The two peaks are the dominate features of the crystalline structure of 3*C*-SiC, and correspond to the modes of transverse (TO) and longitudinal optical (LO) phonons, respectively.[10-12] Compared to those of the pure SiCNPs, these two peaks of P-doped SiCNPs display small blue shifts, which can be ascribed to the quantum confinement effects and the existence of stresses in the nanoparticles induced by the P atoms substituted Si ones

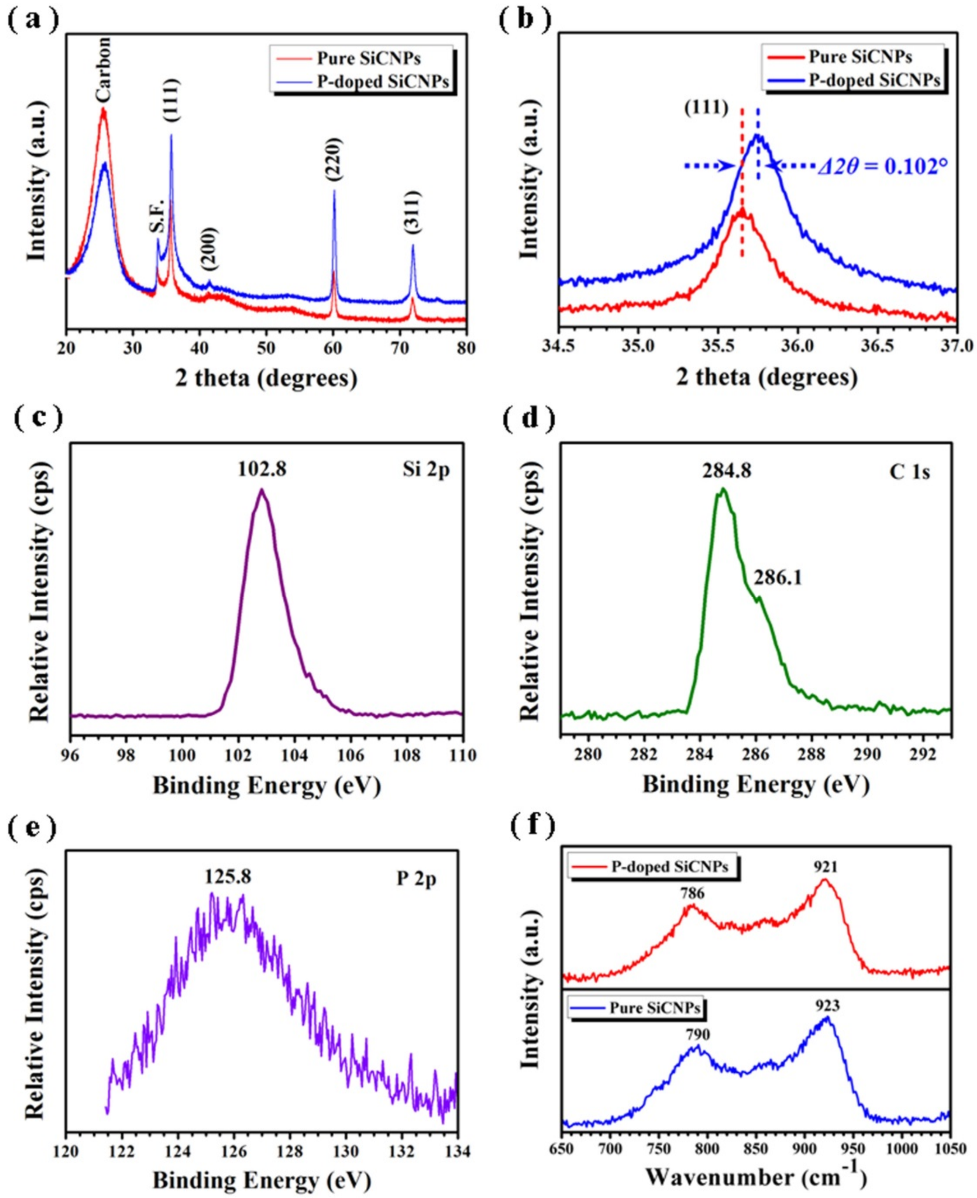

Figure S4. (a) XRD patterns recorded from the pure and P-doped SiCNPs; (b) An enlarged XRD pattern showing the (111) peaks of pure and P-doped SiCNPs; (c) Si 2p binding energy spectrum; (d) C 1s binding energy spectrum; (e) P 2p binding energy spectrum; (f) The Raman scattering spectra of the pure and P-doped SiCNPs.

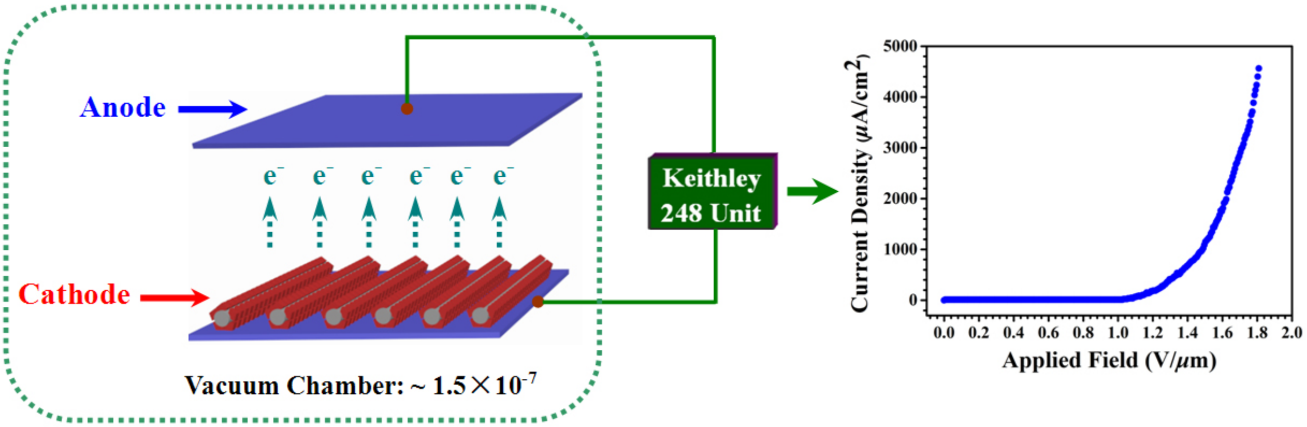

Figure S5. Schematic diagram of the experimental setup used for the measurements of FE properties of SiCNPs field emitters.

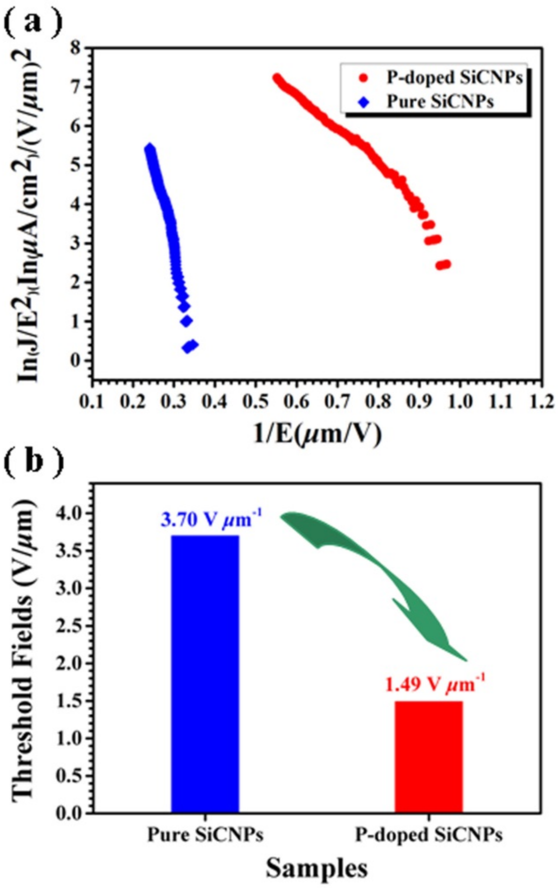

Figure S6. (a) Corresponding Fowler-Nordheim (*F-N*) plots of pure and P-doped SiCNPs under RT. (b) The variation of E_{thr} of pure SiCNPs and P-doped counterparts.

Table S1. Turn-on fields, threshold fields[a], field enhancement factors (*β*), maximum field emission current density and field emission current stability (current density, testing time and current fluctuation) for 1D SiC nanostructured field emitters, other typical nanostructured flexible emitters as well as other commonly used emitters.

	Field emitters	E_{to} (Vμm^{-1})	E_{thr} (Vμm^{-1})	β	Maximum FE current density	Current emission stability	Ref.
1D SiC field emitters	P-doped SiC nanoparticles	0.73-1.03	1.09-1.49	5508	6.6 *m*Acm^{-2}	2.65 *m*Acm^{-2}, 20 h, ±2.1~3.4%	This Work
	N-doped SiC nanoneedles	1.11	1.55	--	~3.5 *m*Acm^{-2}	1.138 *m*Acm^{-2}, 1 h, 8.1%	[13]
	B-doped 3*C*-SiC nanowires	1.35	1.70	4895	~1.45 *m*Acm^{-2}	--, 10 h, 14%	[4]
	N-doped SiC nanoneedles	0.67-1.37	0.96-1.87	2486	~3.5 *m*Acm^{-2}	--, 1 h, 7.7%~14.1%	[14]
	N-doped nanoporous SiC	4.4-9.6	--	936-3636	6000 *m*Acm^{-2}	--, --, --	[15]
	N-doped SiC nanoarrays	1.9-2.65	2.53-3.51	1710	~2.1 *m*Acm^{-2}	--, --, --	[16]
	N-doped 3*C*-SiC nanoneedles	~1.1	--	6500	~5 *m*Acm^{-2}	1.7 *m*Acm^{-2}, 0.5 h, --	[17]
	Tapered SiC nanowires	1.2	--	3368	~0.65 *m*Acm^{-2}	--, --, --	[18]
	Al_2O_3-decorated tublar SiC	2.4	5.37 (10 *m*Acm^{-2})	--	~11 *m*Acm^{-2}	--, --, --	[19]
	Tubular *β*-SiC	5	10 (10 *m*Acm^{-2})	--	~300 *m*Acm^{-2}	--, --, --	[20]
	Aligned SiC porous nanowires	2.3-2.9	--	5241	~2.3 *m*Acm^{-2}	0.57 *m*Acm^{-2}, 20 h, --	[21]
	β-SiC nanowires	--	4	2000	--	--, --, --	[22]
	β-SiC nanoarchitectures	12	--	--	~0.036 *m*Acm^{-2}	--, --, --	[23]
	Bamboo-like *β*-SiC nanowires	10.1	--	--	~0.0085 *m*Acm^{-2}	--, --, --	[24]
	SiC nanowire/nanorods	3.33	5.77 (10 *m*Acm^{-2})	--	--	--, --, --	[25]
	Nonaligned SiC nanowires	3.1-3.5	--	--	~0.09 *m*Acm^{-2}	0.06 *m*Acm^{-2}, 2 h , ±15%	[26]
Other typical	Oriented CuO nanoknife arrays	0.9	--	2400-5400	~1.7 *m*Acm^{-2}	1.15 *m*Acm^{-2}, ~1 h, ~5%	[27]
	Graphene nanosheets	2.04	3.09	--	~1.2 *m*Acm^{-2}	~0.7 *m*Acm^{-2}, 12 h, --	[28]
	Vertical ZnO nanowire/graphene	2.0-2.8	--	3834-6473	~0.85 *m*Acm^{-2}	--, --, --	[29]
	Multiwall carbon nanotubes	2.05 (1 μAcm^{-2})	2.2 (10 μAcm^{-2})	1023, 16434	~0.06 *m*Acm^{-2}	--, 10 h, --	[30]

flexible	Conical nanocarbon	9.5	--	1020	~0.15 *m*Acm^{-2}	--, --, --	[31]
emitters	Tungsten oxide nanowires	--	4.3 (10 *m*Acm^{-2})	1657	~11 *m*Acm^{-2}	5.25 *m*Acm^{-2}, 1 h, ~5%	[32]
	Carbon nanofibers	--	~3.65 (1 μAcm^{-2})	--	~1 *m*Acm^{-2}	0.718 *m*Acm^{-2}, 16 h, --	[33]
	Carbon nanotubes	3.6	--	1112-1546	--	--, --, --	[34]
	Ultrathin single-crystal ZnO nanobelts	--	8.5	700	40.17 *m*Acm^{-2}	7.4 *m*Acm^{-2}, 16 h, ~14%	[35]
	Aligned untralong ZnO nanobelts	1.3	2.9	14000	1.2 *m*Acm^{-2}	--,--,--	[36]
	Carbon nanotubes arrays	--	3	--	10 *m*Acm^{-2}	0.5 *m*Acm^{-2}, 20 h, --	[37]
	Vertical carbon nanotubes	0.4-1.1	--	9000-14500	~1.2 *m*Acm^{-2}	~0.5 *m*A/cm^2, 158 h, --	[38]
Other	Single-layer graphene films	2.3	5.2 (10 *m*Acm^{-2})	3700	23 *m*A/cm^2	11.46 *m*A/cm^2, 12 h, ~4%	[39]
commonly	Single-crystalline PrB_6 nanorods	0.95-2.8	3.55-6.99	823-1390	13.8 *m*A/cm^2	0.025 *m*Acm^{-2}, ~17 h, ＜10%	[40]
used	Single-crystalline LaB_6 nanowires	1.06-1.82	1.62-2.48	1072	5.5 *m*A/cm^2	~0.5 *m*Acm^{-2}, ~17 h, ＜6.0%	[41]
emitters	Ultrafine ZnS nanobelts	3.47	--	2000	11.5 *m*A/cm^2	--,--,--	[42]
	Aligned AlN nanorods	3.8	7	950	~6.7 *m*A/cm^2	--,--,--	[43]
	SnO_2 nanowires	3.5	4.63	1225	~6.5 *m*A/cm^2	~1 *m*Acm^{-2}, 40 h, --	[44]
	Single-crystalline CdS nanobelts	3.7	9.3	1298	~3.5 *m*A/cm^2	~0.2 *m*Acm^{-2}, 75 h, 5%	[45]
	Single-crystalline GaN nanocolumns	2.5	4.7	--	~3.3 *m*A/cm^2	0.003 *m*Acm^{-2}, 1 h, ＜7.4%	[46]

[a] The turn-on and threshold fields required to generate an emission current density of 10 μAcm^{-2} and 1 *m*Acm^{-2}, respectively. If other values are used, it will be mentioned separately.

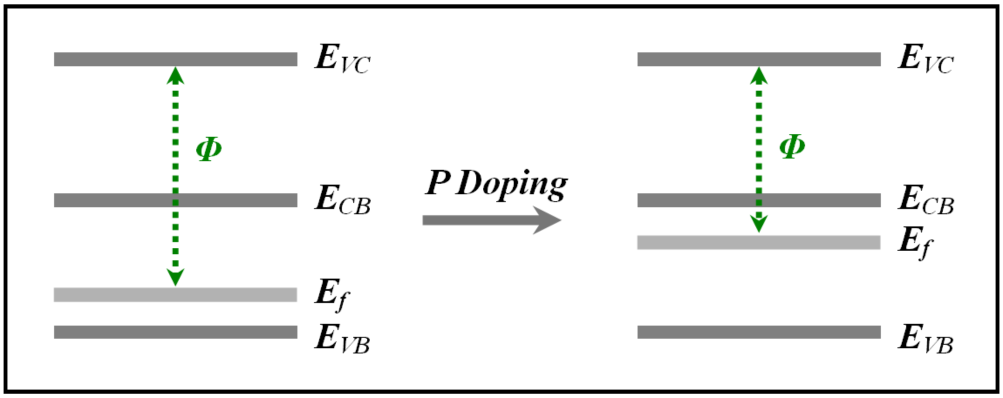

Figure S7 Schematic energy level diagrams of pure and P-doped SiCNPs.

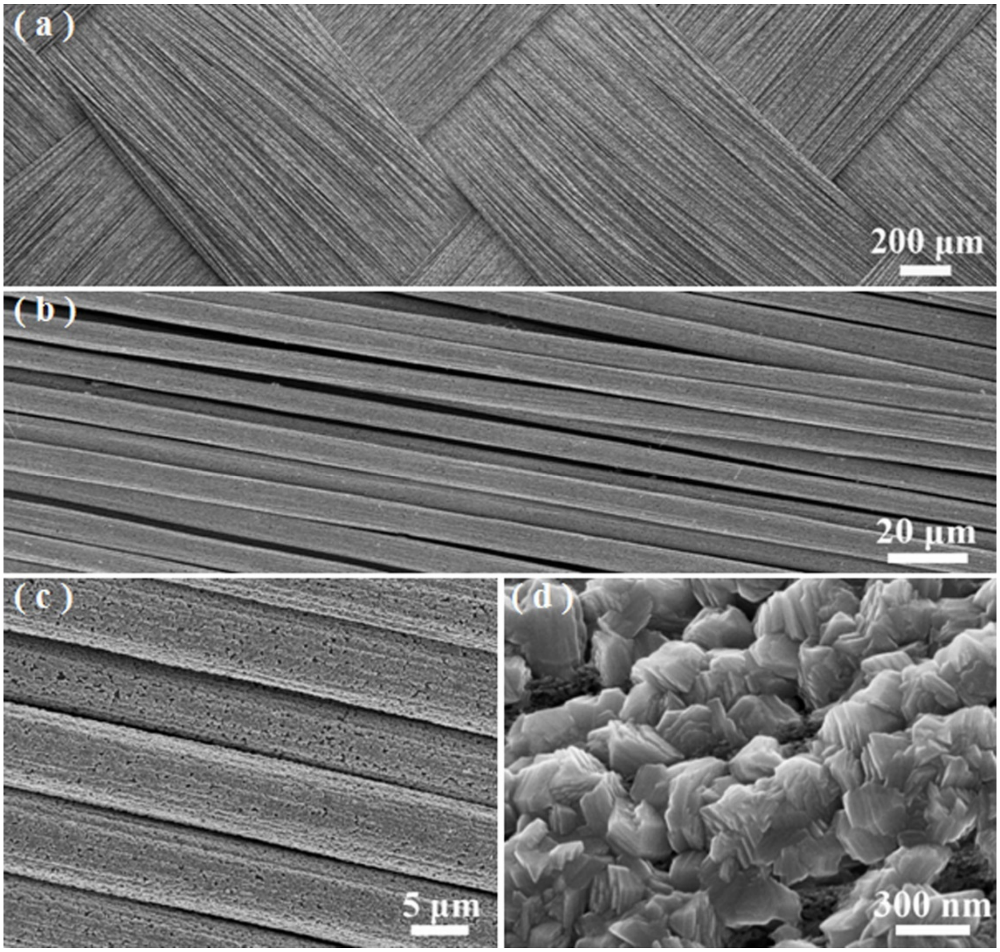

Figure S8. Typical SEM images of the P-doped SiCNPs field emitters under different magnifications after bending for 200 cycles, showing there is nearly no structure damage caused by the bending.

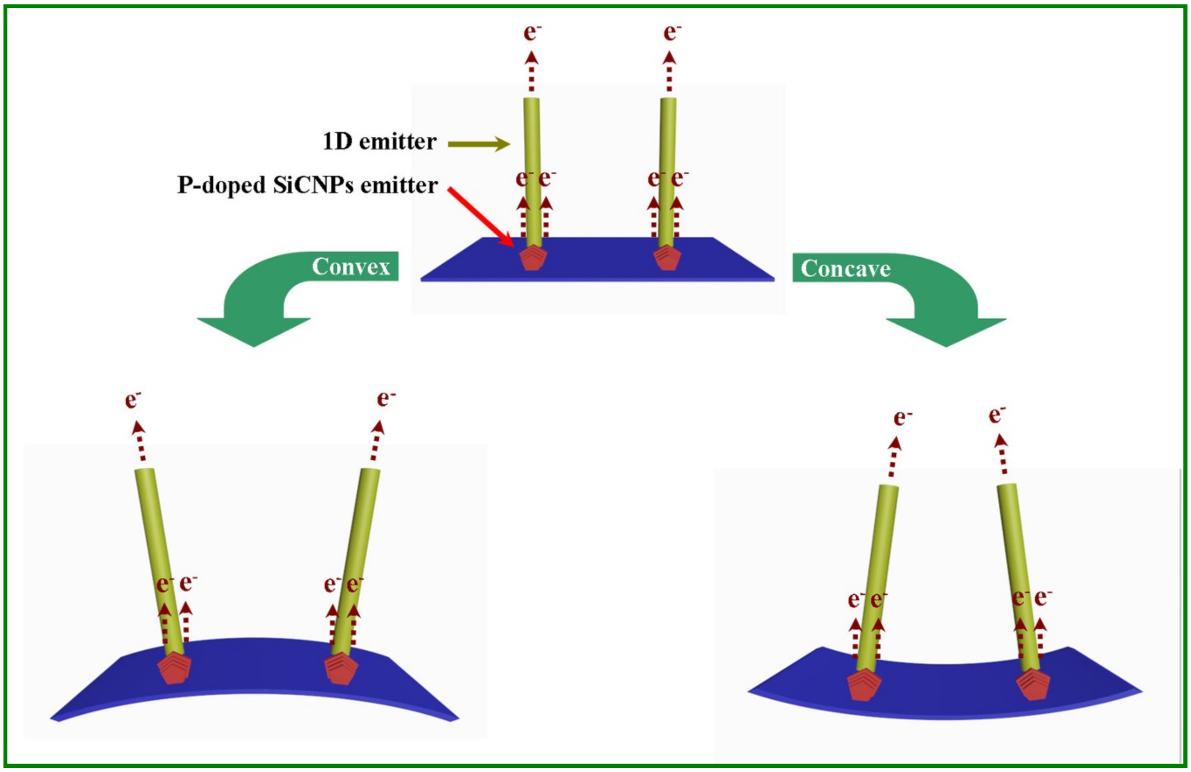

Figure S9. Schematic illustration of the aspect-ratio-depended electron emission directions of the emitters in regard to the bending. It suggests that, as compared to the SiCNPs, the 1D nanomaterials with a high aspect ratio could make a significant change of the electron emission directions caused by the bending of the field emitters. In another word, the SiCNPs with a low aspect ratio is superior to the 1D nanostructures to be applied as the flexible field emitters, since they can greatly limit the "screening effects".

References

[1] A. Dhamne, W. Xu, B. G. Fookes, Y. Fan, L. Zhang, S. Burton, J. Hu, J. Ford, L. An, *J. Am. Ceram. Soc.* **2005**, *88*, 2415.

[2] Y. Li, Y. Liang, Z. Hu, *Ceram. Int.* **1995**, *21*, 59.

[3] F. Gao, W. Feng, G. Wei, J. Zheng, M. Wang, W. Yang, *CrystEngComm* **2012**, *14*, 488.

[4] Y. Yang, H. Yang, G. Wei, L. Wang, M. Shang, Z. Yang, B. Tang, W. Yang, *J. Mater. Chem. C* **2014**, *2*, 4515.

[5] F. Gao, W. Yang, H. Wang, Y. Fan, Z. Xie, L. An, *Cryst. Growth Des.* **2008**, *8*, 1461.

[6] Z. He, L. Wang, F. Gao, G. Wei, J. Zheng, X. Cheng, B. Tang, W. Yang, *CrystEngComm* **2013**, *15*, 2354.

[7] A. Q. Wu, Q. G. Song, L. Yang, *Adv. Mater. Res.* **2012**, *510*, 747.

[8] K. Koumoto, S. Takeda, C. H. Pai, T. Sato, H. Yanagida, *J. Am. Ceram. Soc.* **1989**, *72*, 1985.

[9] R. Iwanowski, K. Fronc, W. Paszkowicz, M. Heinonen, *J. Alloy. Compd.* **1999**, *286*, 143.

[10] Y. Chen, X. Zhang, Q. Zhao, L. He, C. Huang, Z. Xie, *Chem. Commun.* **2011**, *47*, 6398.

[11] J. J. Niu, J. N. Wang, *J. Phys. Chem. B* **2007**, *111*, 4368.

[12] W. Zhou, X. Liu, Y. Zhang, *Appl. Phys. Lett.* **2006**, *89*, 223124.

[13] S. Chen, P. Ying, L. Wang, G. Wei, F. Gao, J. Zheng, M. Shang, Z. Yang, W. Yang, T. Wu, *NPG Asia Mater.* **2015**, *7*, e157.

[14] S. Chen, P. Ying, L. Wang, G. Wei, W. Yang, *Appl. Phys. Lett.* **2014**, *105*, 133106.

[15] M. G. Kang, H. J. Lezec, F. Sharifi, *Nanotechnology* **2013**, *24*, 065201.

[16] S. Chen, P. Z. Ying, L. Wang, G. Wei, J. Zheng, F. Gao, S. Su, W. Yang, *J. Mater. Chem. C* **2013**, *1*, 4779.

[17] X. Zhang, Y. Chen, W. Liu, W. Xue, J. Li, Z. Xie, *J. Mater. Chem. C* **2013**, *1*, 6479.

[18] R. Wu, K. Zhou, J. Wei, Y. Huang, F. Su, J. Chen, L. Wang, *J. Phys. Chem. C* **2012**, *116*, 12940.

[19] H. Cui, L. Gong, G. Yang, Y. Sun, J. Chen, C. Wang, *Phys. Chem. Chem. Phys.* **2011**, *13*, 985.

[20] H. Cui, Y. Sun, G. Yang, J. Chen, D. Jiang, C. Wang, *Chem. Commun.* **2009**, 6243.

[21] Y. Yang, G. Meng, X. Liu, L. Zhang, Z. Hu, C. He, Y. Hu, *J. Phys. Chem. C* **2008**, *112*, 20126.

[22] D. W. Kim, Y. J. Choi, K. J. Choi, J. G. Park, J. H. Park, S. M. Pimenov, V. D. Frolov, N. P. Abanshin, B. I. Gorfinkel, N. M. Rossukanyi, *Nanotechnology* **2008**, *19*, 225706.

[23] G. Shen, Y. Bando, D. Golberg, *Cryst. Growth Des.* **2007**, *7*, 35.

[24] G. Shen, Y. Bando, C. Ye, B. Liu, D. Golberg, *Nanotechnology* **2006**, *17*, 3468.

[25] S. Deng, Z. Li, W. Wang, N. Xu, J. Zhou, X. Zheng, H. Xu, J. Chen, J. She, *Appl. Phys. Lett.* **2006**, *89*, 023118.

[26] W. Zhou, Y. Wu, E. S.-W. Kong, F. Zhu, Z. Hou, Y. Zhang, *Appl. Surf. Sci.* **2006**, *253*, 2056.

[27] S. Das, S. Saha, D. Sen, U. K. Ghorai, D. Banerjee, K. K. Chattopadhyay, *J. Mater. Chem. C* **2014**, *2*, 1321.

[28] H. J. Jeong, H. D. Jeong, H. Y. Kim, S. Y. Jeong, J. T. Han, G. W. Lee, *Small* **2013**, *9*, 2182.

[29] J. O. Hwang, D. H. Lee, J. Y. Kim, T. H. Han, B. H. Kim, M. Park, K. No, S. O. Kim, *J. Mater. Chem.* **2011**, *21*, 3432.

[30] I. Lahiri, V. P. Verma, W. Choi, *Carbon* **2011**, *49*, 1614.

[31] P. Ghosh, M. Z. Yusop, S. Satoh, M. Subramanian, A. Hayashi, Y. Hayashi, M. Tanemura, *J. Am. Chem. Soc.* **2010**, *132*, 4034.

[32] X. Zhang, L. Gong, K. Liu, Y. Cao, X. Xiao, W. Sun, X. Hu, Y. Gao, J. Chen, J. Zhou, *Adv. Mater.* **2010**, *22*, 5292.

[33] H. Sim, S. Lau, H. Yang, L. Ang, M. Tanemura, K. Yamaguchi, *Appl. Phys. Lett.* **2007**, *90*, 143103.

[34] B. J. Yoon, E. H. Hong, S. E. Jee, D. M. Yoon, D. S. Shim, G. Y. Son, Y. J. Lee, K. H. Lee, H. S. Kim, C. G. Park, *J. Am. Chem. Soc.* **2005**, *127*, 8234.

[35] G. Z. Xing, X. S. Fang, Z. Zhang, D. D. Wang, X. Huang, J. Guo, L. Liao, Z. Zheng, H. R. Xu, T. Yu, Z. X. Shen, C. H. A. Huan, T. C. Sum, H. Zhang, T. Wu,

[36] W. Z. Wang, B. Q. Zeng, J. Yang, B. Poudel, J. Huang, M. J. Naughton, Z. Ren, Adv. Mater. 2006, 18, 3275.

[37] S. Fan, M. G. Chapline, N. R. Franklin, T. W. Tombler, A. M. Cassell, H. Dai, Science 1999, 283, 512.

[38] D. H. Lee, J. E. Kim, T. H. Han, J. W. Hwang, S. Jeon, S. Choi, S. H. Hong, W. J. Lee, R. S. Ruoff, S. O. Kim, Adv. Mater. 2010, 22, 1247.

[39] Z. Wu, S. Pei, W. Ren, D. Tang, L. Gao, B. Liu, F. Li, C. Liu, H. Cheng, Adv. Mater. 2009, 21, 1756.

[40] Q. Y. Zhang, J. Q. Xu, Y. M. Zhao, X. H. Ji, S. P. Lau, Adv. Funct. Mater. 2009, 19, 742.

[41] J. Xu, G. Hou, H. Li, T. Zhai, B. Dong, H. Yan, Y. Wang, B. Yu, Y. Bando, D. Golberg, NPG Asia Mater. 2013, 5, e53.

[42] X. S. Fang, Y. Bando, G. Shen, C. Ye, U. K. Gautam, P. M. Costa, C. Zhi, C. Tang, D. Golberg, Adv. Mater. 2007, 19, 2593.

[43] J. H. He, R. Yang, Y. L. Chueh, L. J. Chou, L. J. Chen, Z. L. Wang, Adv. Mater. 2006, 18, 650.

[44] X. S. Fang, J. Yan, L. Hu, H. Liu, P. S. Lee, Adv. Funct. Mater. 2012, 22, 1613.

[45] L. Li, P. Wu, X. S. Fang, T. Zhai, L. Dai, M. Liao, Y. Koide, H. Wang, Y. Bando, D. Golberg, Adv. Mater. 2010, 22, 3161.

[46] Z. Chen, C. Cao, W. S. Li, C. Surya, Crys. Growth Des. 2008, 9, 792.

[47]
